# MicroRNA-125b-5p Drives MMP-2 Expression via Activation of RAGE-38MAPK-p65/p50NF-κB Axis: A Novel Mechanism in Human Lung Cancer Cells

**DOI:** 10.3390/ijms26209983

**Published:** 2025-10-14

**Authors:** Yusuf Saleem Khan, Aisha Farhana, Mohammed Kuddus, Syed Monowar Alam Shahid, Abdullah Alsrhani, Abuzar Abdulwahab Osman, Ghorashy E. Y. Mohammed, Muhammad Ikram Ullah, Zafar Rasheed

**Affiliations:** 1 Department of Anatomy, College of Medicine, University of Hail, Hail 55476, Saudi Arabia; y.salem@uoh.edu.sa; 2 Department of Clinical Laboratory Sciences, College of Applied Medical Sciences, Jouf University, Sakaka 72388, Saudi Arabia; afalserhani@ju.edu.sa (A.A.); mikramullah@ju.edu.sa (M.I.U.); 3 Department of Biochemistry, College of Medicine, University of Hail, Hail 55476, Saudi Arabia; mkuddus@gmail.com (M.K.); sm.shahid@uoh.edu.sa (S.M.A.S.); 4 Department of Pharmacology, College of Medicine, University of Hail, Hail 55476, Saudi Arabia; a.osman@uoh.edu.sa; 5 Department of Pathology, College of Medicine, University of Hail, Hail 55476, Saudi Arabia; g.eltaybe@uoh.edu.sa; 6 Department of Pathology, College of Medicine, Qassim University, P.O. Box 6655, Buraidah 51452, Saudi Arabia; zafarrasheed@qu.edu.sa

**Keywords:** lung cancer, microRNA, matrix metalloproteinase-2, RAGE, S100A4, A549 cells, miR-125b-5p, p38-MAPK, NF-κB, post translational regulation

## Abstract

Dysregulated microRNA-mediated control of matrix metalloproteinase-2 (MMP-2) plays a pivotal role in lung cancer (LC) progression, though the inflammatory signaling mechanisms governing its regulation remain poorly understood. This study reveals how S100A4-activated RAGE signaling modulates MMP-2 expression through microRNA-125b-5p (miR-125b-5p) in human LC cells. Potential miRNA target genes were computationally predicted using TargetScan algorithms. Functional interaction between miR-125b-5p and MMP-2 3′UTR was experimentally validated through dual-luciferase reporter assays incorporating full-length MMP-2 3′UTR sequence. Further validation was performed through transfection with miRNA inhibitors or mimics. To delineate the underlying mechanisms, key pathways were inhibited using small-molecule antagonists targeting p38-MAPK and NF-κB. Our analysis identified a conserved miR-125b-5p binding site in the MMP-2 3′UTR. In A549 cells, S100A4 induced reciprocal regulation, simultaneously upregulating MMP-2 and downregulating miR-125b-5p, with luciferase assays confirming direct targeting. Pre-miR-125b-5p transfection effectively reduced endogenous MMP-2 levels, while p38-MAPK/NF-κB activation mediated this regulation by suppressing miR-125b-5p consequently elevating MMP-2 expression. These findings were further validated in another human LC cell, SHP-77. These findings provide the first evidence demonstrating that miR-125b-5p directly regulates MMP-2 in LC, establishing S100A4-RAGE⟶p38/NF-κB⟶miR-125b-5p⟶MMP-2 axis as a novel regulatory pathway. The results position miR-125b-5p as a dual-action biomarker and therapeutic target against MMP-2-driven LC metastasis, offering new insights into critical inflammation-to-cancer connections.

## 1. Introduction

Lung cancer (LC) is one of the most prevalent and lethal malignancies worldwide, accounting for a significant proportion of cancer-related deaths. Despite advances in early detection and therapeutic interventions, the prognosis for LC remains poor, primarily due to its aggressive nature and high metastatic potential [1]. Understanding the molecular mechanisms that drive lung cancer progression is crucial for developing novel therapeutic strategies. One of the key players in this process is the matrix metalloproteinase (MMP) family, particularly MMP-2, which plays a vital role in extracellular matrix (ECM) degradation, facilitating tumor cell invasion and metastasis [2,3]. MMP-2, also known as gelatinase A, has been widely studied for its involvement in tumor microenvironment remodeling. Its upregulation is often associated with poor clinical outcomes and increased invasiveness of cancer cells [4,5]. The regulation of MMPs including MMP-2 is complex, involving transcriptional, post-transcriptional, and post-translational mechanisms. Among these, post-transcriptional regulation via microRNAs (miRNAs) has emerged as a critical factor in controlling MMP-2 expression [6]. MiRNAs are short non-coding RNAs that regulate gene expression by associating with the 3′ untranslated region (3′UTR) of target mRNAs, causing mRNA degradation or translational repression. Recent research has emphasized the importance of microRNA-125b-5p (hsa-miR-125b-5p) as a key regulatory molecule in various human disorders, including lung cancers [7,8,9]. MiR-125b-5p has been identified as a tumor suppressor in numerous cancers, where it modulates crucial pathways involved in cell proliferation, apoptosis, and metastasis [10,11]. One of the significant contributors to lung cancer progression is the receptor for advanced glycation end products (RAGE), which is activated by its ligand S100A4 [12]. RAGE signaling activated by S100A4 has been implicated in various inflammatory and oncogenic processes, including tumor growth, metastasis, and resistance to apoptosis [13,14]. Interestingly, recent analyses of TCGA data show that AGER (encoding RAGE) expression is significantly downregulated in lung adenocarcinoma compared to matched normal lung tissue, and that low RAGE expression correlates with poorer overall and disease-free survival [15].

Several studies also report that reduced RAGE mRNA and protein levels are associated with more advanced tumor stage (TNM stage) in non-small cell lung carcinoma, regardless of histological subtype [16]. S100A4, a member of the S100 protein family, has been demonstrated to enhance MMP-2 expression [17,18].

The downstream signaling pathways of RAGE activation involve several key molecules, including mitogen-activated protein kinases (MAPKs) and nuclear factor-kappa B (NF-κB). These molecules play crucial roles in cancer cell survival, proliferation, and metastasis [19,20].

MAPKs such as p38-MAPK, c-Jun N-terminal kinase (JNK), and extracellular signal-regulated kinase (ERK) are integral to cellular responses to external stimuli. These kinases regulate various cellular processes, such as differentiation, proliferation, and apoptosis [21]. In the context of lung cancer, MAPKs have been particularly implicated in promoting MMPs [2]. Studies also suggest that MAPKs activation leads to the suppression of tumor-suppressive miRNAs, thereby enhancing oncogenic signaling [22]. Similarly, NF-κB, associated with inflammation and cancer, has been shown to regulate MMPs expression through complex feedback mechanisms [23,24]. In this work, we investigate the interaction between hsa-miR-125b-5p and MMP-2 in lung cancer cells A549 and SHP-77, with a particular emphasis on the effect of RAGE activation via S100A4. We hypothesize that S100A4-mediated RAGE signaling downregulates hsa-miR-125b-5p expression, leading to increased MMP-2 levels through activation of the MAPKs and NF-κB pathways. To test this hypothesis, we employed a combination of bioinformatics predictions, luciferase reporter assays, and functional analyses using miRNA mimics and inhibitors.

## 2. Results

### 2.1. Bioinformatics Prediction of hsa-miR-125b-5p Binding to the 3′UTR of MMP-2 mRNA (ENST00000219070.4)

**Figure 1:**
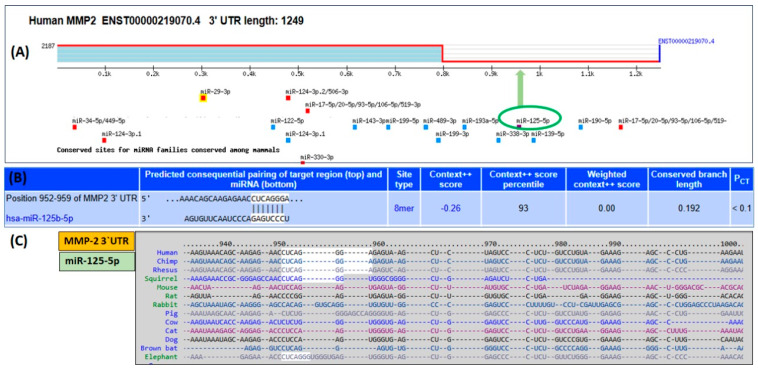
Bioinformatics analysis of hsa-miR-125b-5p binding to the 3′UTR of human MMP-2 mRNA using the TargetScan algorithm. (**A**) Identification of seed-matching sequences for hsa-miR-125b-5p (highlighted in a green elliptical circle) within the 3′UTR of MMP-2 mRNA. (**B**) Predicted base-pairing interactions between hsa-miR-125b-5p and the seed region of MMP-2 mRNA’s 3′UTR, including duplex structure, site type, context score, percentile ranking, weighted context score, conserved branch length, and probability of conserved targeting (PCT). (**C**) Evolutionary conservation of the hsa-miR-125b-5p seed region within the 3′UTR of MMP-2 across multiple species. Note: The “++” signifies a more advanced and accurate prediction model compared to the initial context score.

The TargetScan algorithm was used to determine putative hsa-miR-125b-5p binding sites within the 3′ untranslated region (3′UTR) of human MMP-2 mRNA (ENST00000219070.4). The analysis revealed that the MMP-2 3′UTR, which is 1249 nucleotides long, has conserved binding sequences for multiple miRNAs, including hsa-miR-125b-5p (Figure 1A). Specifically, hsa-miR-125b-5p was predicted to interact with nucleotides 952–959 of the MMP-2 3′UTR through an 8-mer site type. The computational analysis further provided the following parameters: context score (−0.26), context score percentile (93), weighted context score (0.00), conserved branch length (0.192), and probability of conserved targeting (PCT < 0.1). These predicted interactions are illustrated in Figure 1B. Furthermore, evolutionary conservation analysis using TargetScan identified similar binding sequences for hsa-miR-125b-5p in multiple species, including chimpanzees, rhesus monkeys, and squirrels, suggesting a conserved regulatory mechanism across species (Figure 1C).

### 2.2. Relationship Between hsa-miR-125b-5p and MMP-2 in RAGE-Mediated S100A4 Stimulation of Human Lung Cancer Cells

**Figure 2:**
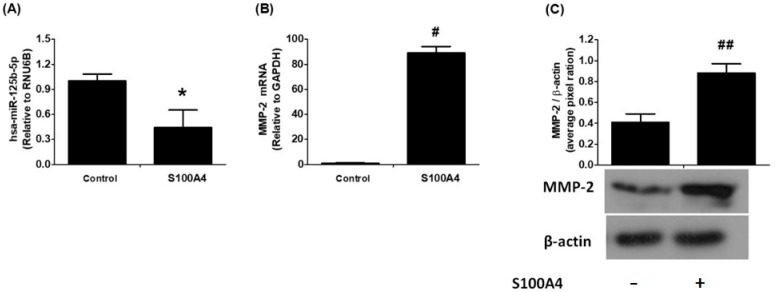
Negative correlation between hsa-miR-125b-5p and MMP-2 in S100A4-induced RAGE signaling in human lung cancer cells. (**A**) Quantification of hsa-miR-125b-5p expression in A549 lung cancer cells following stimulation with S100A4, measured using TaqMan assays. * *p* < 0.01 compared to the control group. Unstimulated A549 cells served as controls, with RNU6B used as the internal reference gene. (**B**) Analysis of MMP-2 mRNA levels in A549 cells after S100A4 stimulation, assessed via TaqMan assays. GAPDH was used as the endogenous control. # *p* < 0.0001 compared to the control. (**C**) Detection of MMP-2 protein secretion in the culture medium of S100A4-treated A549 cells using Western blot analysis. Densitometric analysis was performed on band intensities. β-Actin was used as a loading control. ## *p* < 0.01 compared to the control. Serum-starved A549 cells were treated with S100A4 (1 µg/mL) for 24 h. Data are presented as mean ± SEM from five independent experiments.

To investigate the effects of S100A4 protein on RAGE signaling, the expression levels of hsa-miR-125b-5p and MMP-2 mRNA were measured in A549 human lung cancer cells using highly specific quantitative RT-PCR with TaqMan assays. Following 24 h of S100A4 treatment, a significant reduction in hsa-miR-125b-5p expression was observed (Figure 2A; *p* < 0.05). Notably, this decrease in hsa-miR-125b-5p was negatively linked with an upregulation of MMP-2 mRNA levels (Figure 2B; *p* < 0.001). Furthermore, the analysis of cell culture supernatants showed a significant increase in MMP-2 protein production in response to S100A4 stimulation (Figure 2C). These findings indicate that S100A4-induced activation of RAGE signaling leads to hsa-miR-125b-5p downregulation, which may contribute to the enhanced MMP-2 production and secretion in lung cancer cells.

### 2.3. Validation of hsa-miR-125b-5p Interatcion to the Predicted 3′UTR Target Site of MMP-2 mRNA

**Figure 3:**
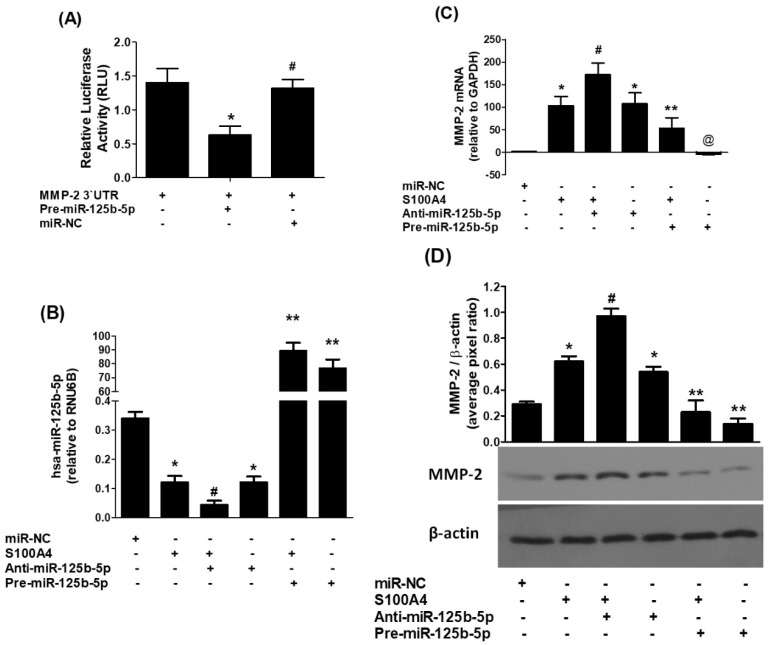
Direct suppression of MMP-2 expression by hsa-miR-125b-5p in human lung cancer cells. (**A**) Luciferase assay results showing the activity in A549 cells transfected with a reporter vector and pre-miR-125b-5p. Cells transfected with a microRNA negative control (miR-NC) served as a negative control. * *p* < 0.05 versus #. (**B**) Quantification of hsa-miR-125b-5p levels in A549 cells transfected with either anti-miR-125b-5p or pre-miR-125b-5p following 24 h stimulation with S100A4 (1 µg/mL). miR-NC-transfected cells were used as a negative control, with RNU6B as the internal reference gene. * *p* < 0.05 versus miR-NC; # *p* < 0.05 versus*; # *p* < 0.0001 versus **. (**C**) Measurement of MMP-2 mRNA levels in A549 cells transfected with anti-miR-125b-5p or pre-miR-125b-5p and treated with S100A4 for 24 h. miR-NC-transfected cells served as a negative control, and GAPDH was used as the internal reference. * *p* < 0.001 versus miR-NC; # *p* < 0.05 versus *; # *p* < 0.001 versus **; ** *p* < 0.001 versus @. (**D**) Western blot analysis of MMP-2 protein levels in the culture medium of A549 cells transfected with anti-miR-125b-5p or pre-miR-125b-5p following 24 h S100A4 stimulation. miR-NC-transfected cells served as a negative control, with β-actin as the loading control. * *p* < 0.001 versus miR-NC; # *p* < 0.05 versus *; # *p* < 0.001 versus **. Each bar in the histograms represents the mean ± SEM from five independent experiments.

To determine whether hsa-miR-125b-5p directly targets the 3′UTR of MMP-2 mRNA, a luciferase reporter assay was conducted with a construct harboring the full-length 3′UTR of MMP-2 (NM_004530.6). A549 cells co-transfected with pre-miR-125b-5p showed a substantial decrease in luciferase activity (*p* < 0.01), indicating functional interaction between the miRNA and its predicted binding site. In contrast, cells transfected with a microRNA-negative control (miR-NC) demonstrated no difference in luciferase activity (*p* > 0.05) (Figure 3A). These results confirm that hsa-miR-125b-5p specifically binds to the seed-matched region within the 3′UTR of MMP-2 mRNA, supporting its role in post-transcriptional regulation of MMP-2 expression.

### 2.4. Direct Regulation of MMP-2 Expression by hsa-miR-125b-5p in RAGE-Mediated Lung Cancer Cells

To study the impact of hsa-miR-125b-5p on MMP-2 expression, A549 lung cancer cells were transfected with either anti-miR-125b-5p or pre-miR-125b-5p (100 nM), followed by stimulation with S100A4 (1 µg/mL) or left unstimulated. The results indicated that S100A4 stimulation alone or transfection with anti-miR-125b-5p in the absence of S100A4 significantly reduced hsa-miR-125b-5p expression relative to cells transfected with the negative control (miR-NC) or unstimulated A549 cells (*p* < 0.01). Notably, transfection with anti-miR-125b-5p further suppressed the S100A4-induced expression of hsa-miR-125b-5p (*p* < 0.05). Overexpressing hsa-miR-125b-5p by pre-miR-125b-5p transfection reversed these effects, considerably raising hsa-miR-125b-5p levels in both unstimulated cells and S100A4-treated cells (*p* < 0.001). These findings are summarized in Figure 3B.

To evaluate the effect of hsa-miR-125b-5p on MMP-2 mRNA expression, qRT-PCR analysis was performed. The results demonstrated that either S100A4 stimulation or transfection with anti-miR-125b-5p alone significantly increased MMP-2 expression compared to miR-NC-transfected or unstimulated A549 cells (*p* < 0.001). Furthermore, anti-miR-125b-5p transfection enhanced the S100A4-induced upregulation of MMP-2 (*p* < 0.05). In contrast, pre-miR-125b-5p transfection significantly downregulated MMP-2 expression both in the presence and absence of S100A4 stimulation (*p* < 0.001), as illustrated in Figure 3C. To determine whether these changes at the mRNA level were reflected in protein expression, MMP-2 secretion into the culture medium was assessed using Western blot analysis. Consistent with the mRNA findings, both S100A4 stimulation and anti-miR-125b-5p transfection independently increased MMP-2 protein levels compared to miR-NC-transfected or unstimulated A549 cells (*p* < 0.001). Furthermore, anti-miR-125b-5p further augmented S100A4-induced MMP-2 secretion (*p* < 0.05). In contrast, pre-miR-125b-5p transfection significantly reduced MMP-2 protein levels, both with and without S100A4 treatment (*p* < 0.01), as shown in Figure 3D. These findings provide strong evidence that hsa-miR-125b-5p directly regulates MMP-2 expression and secretion in A549 LC cells, likely through its interaction with the MMP-2 3′UTR. The observed inverse connection between hsa-miR-125b-5p and MMP-2 suggests that this microRNA is critical in modulating MMP-2-mediated processes in lung cancer progression.

### 2.5. Role of p38-MAPKs in S100A4-RAGE-Mediated Modulation of hsa-miR-125b-5p and MMP-2

**Figure 4:**
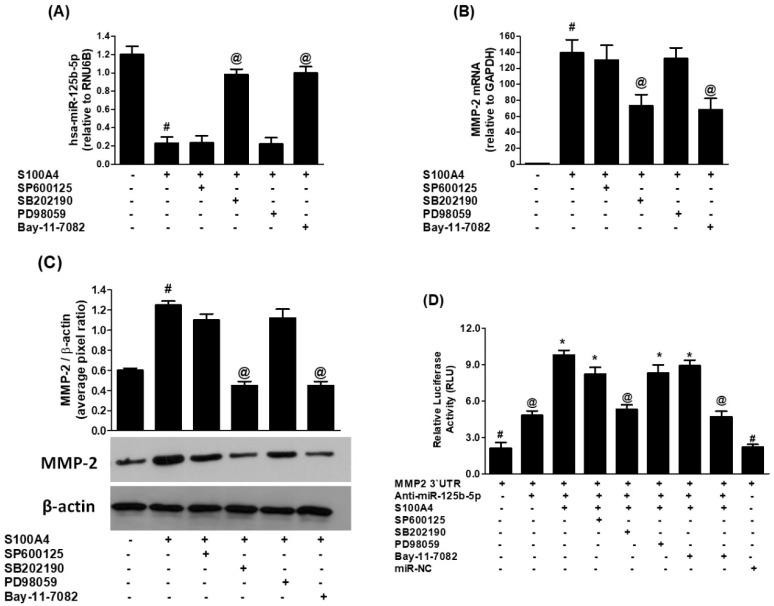
Role of MAPKs and NF-κB pathways in S100A4-RAGE-mediated modulation of hsa-miR-125b-5p and MMP-2 expression. A549 human lung cancer (LC) cells were pretreated for 2 h with specific inhibitors targeting JNK-MAPKs (SP600125, 50 µM), p38-MAPKs (SB202190, 50 µM), ERK-MAPKs (PD98059, 50 µM), or NF-κB (Bay 11-7082, 50 µM), followed by stimulation with S100A4 (1 µg/mL). (**A**) Expression levels of hsa-miR-125b-5p in S100A4-stimulated A549 cells after treatment with MAPKs or NF-κB inhibitors. # *p* < 0.01 versus untreated cells; @ *p* < 0.001 versus #. (**B**) MMP-2 mRNA expression in S100A4-stimulated A549 cells following treatment with MAPKs or NF-κB inhibitors. # *p* < 0.0001 versus untreated cells; @ *p* < 0.01 versus #. (**C**) Western blot analysis of MMP-2 protein levels in the culture medium of S100A4-stimulated A549 cells treated with MAPKs or NF-κB inhibitors. # *p* < 0.001 versus untreated cells; @ *p* < 0.01 versus #. (**D**) Luciferase assay results from A549 cells co-transfected with the MMP-2 3′UTR reporter vector and anti-miR-125b-5p. miR-negative control (miR-NC) was used as a negative control. After 24 h of transfection, cells were pretreated with MAPKs or NF-κB inhibitors for 2 h before S100A4 stimulation for 24 h. # *p* < 0.05 versus @; @ *p* < 0.01 versus *. Each bar in the histograms represents the mean ± SEM from five independent experiments.

We determine whether the modulation of hsa-miR-125b-5p and MMP-2 by S100A4-RAGE signaling in human lung cancer cells (A549) involves the activation of mitogen-activated protein kinases (MAPKs), including p38-MAPK, Jun kinase (JNK/SAPK), and extracellular signal-regulated kinase (ERK) pathways. To explore p38-MAPK, A549 cells were pretreated with the p38-MAPK inhibitor SB202190 (50 µM) for 2 h before stimulation with S100A4 (1 µg/mL) for 24 h. hsa-miR-125b-5p expression levels were quantified using TaqMan assays. Compared to untreated cells, S100A4 stimulation led to a significant downregulation of hsa-miR-125b-5p (Figure 4A, bars 1 & 2; *p* < 0.001). Notably, inhibition of p38-MAPK activity with SB202190 significantly restored hsa-miR-125b-5p expression (Figure 4A, bar 4; *p* < 0.001). Interestingly, the negative relationship between hsa-miR-125b-5p and MMP-2 expression was confirmed, as inhibition of p38-MAPK reversed S100A4-induced upregulation of MMP-2 mRNA (Figure 4B, bar 4; *p* < 0.05). To assess if these transcriptional changes were mirrored in protein levels, MMP-2 secretion into the culture medium was measured using Western blot analysis. As expected, exposure to S100A4 significantly increased MMP-2 protein levels compared to untreated cells (Figure 4C, bars 1 & 2; *p* < 0.01). However, inhibition of p38-MAPK effectively suppressed this S100A4-induced MMP-2 production (Figure 4C, bar 4; *p* < 0.01). The results imply that p38-MAPK activation is critical in regulating MMP-2 expression while simultaneously acting as a negative regulator of hsa-miR-125b-5p in lung cancer cells.

To further validate the involvement of the p38-MAPK pathway in this regulatory mechanism, luciferase reporter assays were performed in A549 cells co-transfected with an MMP-2-3′UTR reporter vector and anti-miR-125b-5p. Following 24 h of transfection, cells were pretreated with SB202190 for 2 h before S100A4 stimulation. The results indicated that S100A4 treatment significantly increased MMP-2-3′UTR luciferase activity in anti-miR-125b-5p-transfected cells (*p* < 0.01). However, pretreatment with the p38-MAPK inhibitor significantly reduced this S100A4-induced luciferase activity (*p* < 0.01). In contrast, control transfections with the MMP-2 3′UTR reporter vector alone or with a negative control microRNA (miR-NC) did not affect luciferase activity, serving as negative controls (Figure 4D, bars 1 & 9; *p* > 0.05). Overall, these findings provide strong evidence that p38-MAPK activation mediates the suppression of hsa-miR-125b-5p while promoting MMP-2 expression in response to S100A4-RAGE signaling in human lung cancer cells. Moreover, the involvement of MMP-2′s 3′UTR in this regulatory pathway highlights its significance in the post-transcriptional regulation of gene expression through p38-MAPK signaling.

### 2.6. Role of JNK-MAPKs and ERK-MAPKs in S100A4-RAGE-Induced Modulation of hsa-miR-125b-5p and MMP-2

To investigate whether S100A4-RAGE signaling influences hsa-miR-125b-5p and MMP-2 expression through JNK-MAPKs or ERK-MAPKs in A549 lung cancer cells, we employed specific MAPK inhibitors. A549 cells were pretreated for 2 h with SP600125 (50 µM, JNK inhibitor) or PD98059 (50 µM, ERK inhibitor) before stimulation with S100A4 (1 µg/mL) for 24 h. As expected, S100A4 stimulation significantly downregulated hsa-miR-125b-5p expression compared to untreated cells (Figure 4A, bars 1 & 2; *p* < 0.001). However, pretreatment with either the JNK inhibitor (SP600125) or the ERK inhibitor (PD98059) did not restore hsa-miR-125b-5p expression, indicating that these pathways do not regulate its suppression (Figure 4A, bars 3 & 5; *p* > 0.05). Similarly, neither inhibitor significantly attenuated S100A4-induced MMP-2 mRNA expression (Figure 4B, bars 3 & 5; *p* > 0.05). To determine whether these transcriptional changes were mirrored in protein levels, MMP-2 secretion was assessed using Western blot analysis. While inhibition of JNK or ERK MAPKs slightly reduced S100A4-induced MMP-2 protein levels, the decrease was not statistically significant (Figure 4C, bands/bars 3 & 4; *p* > 0.05).

To further confirm that JNK and ERK MAPKs are not involved in the regulation of hsa-miR-125b-5p and MMP-2, we performed luciferase reporter assays in A549 cells co-transfected with the MMP-2-3′UTR reporter vector and anti-miR-125b-5p. After 24 h, cells were pretreated with SP600125 or PD98059 for 2 h before S100A4 stimulation. As expected, S100A4 treatment alone significantly increased MMP-2-3′UTR luciferase activity (*p* < 0.01). However, pretreatment with either JNK or ERK inhibitors did not significantly reduce this increase (Figure 4D, bars 4 & 6; *p* > 0.05). Collectively, these findings demonstrate that the S100A4-RAGE-mediated suppression of hsa-miR-125b-5p and upregulation of MMP-2 is specifically regulated via the p38-MAPK pathway, while JNK and ERK MAPKs appear to play no significant role in this process.

### 2.7. Role of NF-κB Transcription Factor in S100A4-RAGE-Induced Modulation of hsa-miR-125b-5p and MMP-2

RAGE-mediated regulation of miRNAs is known to be controlled by NF-κB signaling. Here, we investigated whether NF-κB activation is involved in S100A4-RAGE-induced modulation of hsa-miR-125b-5p and MMP-2 in human A549 lung cancer (LC) cells. To assess this, A549 cells were pretreated with the NF-κB inhibitor Bay-11-7082 (50 µM) for 2 h before stimulation with S100A4 (1 µg/mL) for 24 h. Inhibition of NF-κB significantly upregulated hsa-miR-125b-5p expression (Figure 4A, bar 6; *p* < 0.001). Interestingly, this effect was inversely correlated with MMP-2 expression—NF-κB inhibition significantly attenuated S100A4-induced MMP-2 mRNA levels (Figure 4B, bar 6; *p* < 0.05). At the protein level, Western blot analysis revealed that NF-κB inhibition markedly reduced S100A4-induced MMP-2 secretion in A549 cell culture supernatants (Figure 4D, band/bar 6; *p* < 0.01). These results indicate that NF-κB activation displays dual functions, promoting MMP-2 expression while suppressing hsa-miR-125b-5p in S100A4-stimulated lung cancer cells.

To further confirm NF-κB’s role in this regulatory mechanism, we performed luciferase reporter assays in A549 cells co-transfected with the MMP-2-3′UTR reporter vector and anti-miR-125b-5p. Following 24 h of transfection, cells were pretreated with Bay-11-7082 for 2 h before S100A4 stimulation. As expected, S100A4 significantly increased MMP-2-3′UTR luciferase activity (*p* < 0.01). Notably, inhibiting NF-κB significantly reduced this rise (*p* < 0.01), further supporting NF-κB’s role in S100A4-induced MMP-2 regulation.

**Figure 5:**
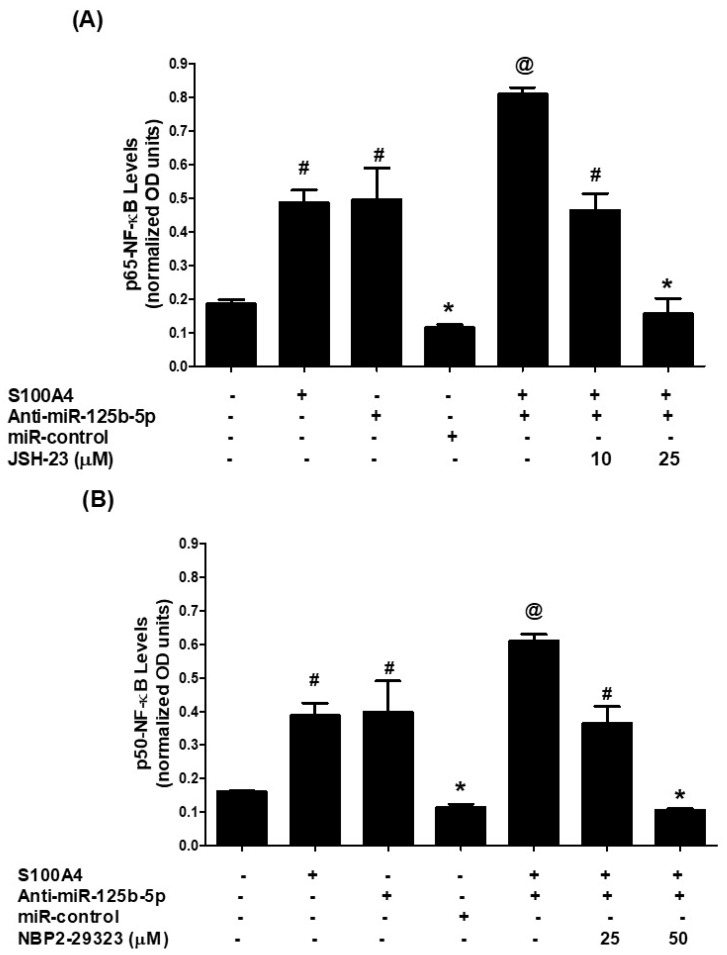
S100A4-RAGE-induced NF-κB activation is regulated by hsa-miR-125b-5p in A549 lung cancer cells transfected or non-transfected with anti-miR-125b-5p. (**A**) Activation of p65-NF-κB in A549 cells, either transfected or non-transfected with anti-miR-125b-5p, following S100A4 stimulation. p65-NF-κB activity was measured using a specific transcription factor assay kit (Abcam). JSH23, a selective p65-NF-κB inhibitor, served as a control for inhibition. # *p* < 0.001 versus untreated cells; * *p* < 0.0001 versus #; @ *p* < 0.05 versus #. (**B**) Activation of p50-NF-κB in A549 cells, either transfected or non-transfected with anti-miR-125b-5p, in response to S100A4 stimulation. p50-NF-κB activity was assessed using a specific transcription factor assay kit (Abcam). NBP2-29323, a selective p50-NF-κB inhibitor, was used as a control for inhibition. # *p* < 0.001 versus untreated cells; * *p* < 0.0001 versus #; @ *p* < 0.05 versus #. Each bar in the histograms represents the mean ± SEM from five independent experiments.

To determine the extent of NF-κB pathway involvement, we analyzed nuclear levels of NF-κB subunits p65 and p50 in A549 cells (non-transfected or transfected with anti-miR-125b-5p) following 30 min of S100A4 stimulation. Nuclear extracts from S100A4-treated cells showed a significant increase in p65 levels compared to untreated controls (Figure 5A, *p* < 0.01). Similarly, anti-miR-125b-5p transfection led to a marked increase in nuclear p65, which was further enhanced upon S100A4 stimulation (*p* < 0.001). The role of p65 in this process was validated using JSH-23, a specific p65 inhibitor. Pretreatment with JSH-23 (10–25 µM) led to a dose-dependent inhibition of S100A4-induced nuclear p65 levels (Figure 5A, bars 6 & 7; *p* < 0.01). Similarly, S100A4 stimulation increased nuclear p50 levels (Figure 5B, *p* < 0.01), and this effect was amplified in anti-miR-125b-5p-transfected cells (*p* < 0.001). Treatment with NBP2-29323, a specific p50 inhibitor, significantly reduced S100A4-induced nuclear p50 levels in a dose-dependent manner (Figure 5B, bars 6 & 7; *p* < 0.01).

### 2.8. Validation of Data Using SHP-77 Lung Cancer Cell Line

**Figure 6:**
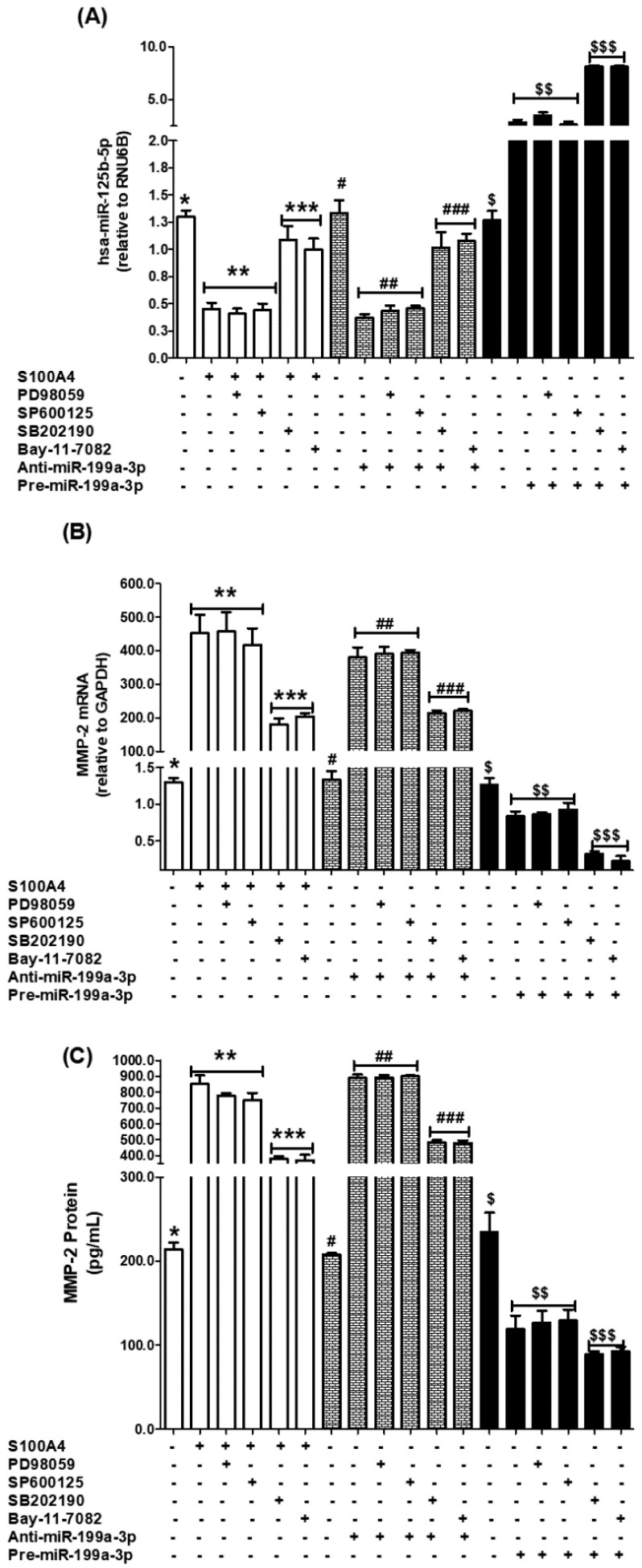
p38-MAPK and NF-κB inhibitors upregulate hsa-miR-125b-5p (**A**) and downregulate MMP-2 mRNA (**B**) and MMP-2 protein secretion (**C**) in SHP-77 human lung cancer cells treated with S100A4, transfected with anti-miR-125b-5p or pre-anti-miR-125b-5p. Expressions of microRNA and mRNA were quantified by TaqMan assays, whereas MMP-2 secretion in the culture medium was determined by MMP-2 highly specific Sandwich ELISA (Abcam). SHP-77 cells were treated with S100A4 or transfected with anti-miR-125b-5p/pre-miR-125b-5p and treated with ERK inhibitor (PD98059), JNK inhibitor (SP600125), p38 inhibitor (SB202190), or NF-κB inhibitor (Bay-11-7082) for 24 h. Data show mean ± SD (* *p* < 0.05 vs. **; * *p* < 0.05 vs. ***; *** *p* < 0.05 vs. **), (# *p* < 0.05 vs. ##; # *p* < 0.05 vs. ###; ### *p* < 0.05 vs. ##), ($ *p* < 0.05 vs. $$; $ *p* < 0.05 vs. $$$; $$$ *p* < 0.05 vs. $$).

The results obtained in A549 lung cancer cells were further validated using another human lung cancer cell line, SHP-77 (Figure 6). To evaluate the contribution of MAPK and NF-κB signaling pathways, SHP-77 cells were pretreated with specific inhibitors—SB202190 (p38), SP600125 (JNK), PD98059 (ERK), and the NF-κB inhibitor Bay11-7082—followed by stimulation with the RAGE pathway inducer, S100A4 protein. Expression levels of miR-125b-5p were quantified by real-time PCR using TaqMan assays (Figure 6A). Treatment with either the p38-MAPK inhibitor or the NF-κB inhibitor significantly upregulated miR-125b-5p expression (*p* < 0.05), whereas inhibition of ERK-MAPK or JNK-MAPK failed to elicit this effect in S100A4-stimulated SHP-77 cells (Figure 6A). To further confirm these findings, SHP-77 cells were transfected with anti-miR-125b-5p and subsequently treated with the same inhibitors. Quantitative PCR analysis again revealed that inhibition of p38-MAPK or NF-κB significantly enhanced miR-125b-5p expression (*p* < 0.05), while ERK-MAPK and JNK-MAPK inhibition did not produce a significant effect (Figure 6A). A similar pattern was observed in SHP-77 cells transfected with pre-miR-125b-5p (mimic), where treatment with p38-MAPK or NF-κB inhibitors significantly increased miR-125b-5p expression (*p* < 0.05), whereas ERK-MAPK and JNK-MAPK inhibitors had no measurable effect (Figure 6A).

Next, the regulatory impact of these pathways on MMP-2 expression was assessed. Pretreatment of SHP-77 cells with p38-MAPK or NF-κB inhibitors prior to S100A4 stimulation significantly suppressed MMP-2 mRNA expression (*p* < 0.05), while inhibition of ERK-MAPK or JNK-MAPK did not alter MMP-2 transcript levels (Figure 6B). These results were further validated in SHP-77 cells transfected with anti-miR-125b-5p or with pre-miR-125b-5p, where inhibition of p38-MAPK or NF-κB again significantly reduced MMP-2 mRNA expression (*p* < 0.05), but ERK-MAPK and JNK-MAPK inhibitors were ineffective (Figure 6B).

Finally, culture supernatants of all sets of treated or transfected SHP-77 cells were analyzed for MMP-2 release using a specific ELISA. Treatment with either p38-MAPK or NF-κB inhibitors markedly decreased extracellular MMP-2 levels in S100A4-stimulated SHP-77 cells (*p* < 0.05), whereas ERK-MAPK and JNK-MAPK inhibition failed to exert a significant effect in the same S100A4-stimulated cells (Figure 6C). The results also showed that the excessive production of MMP-2 by anti-miR-125b-5p-transfected cells was significantly reduced by the treatment with p38-MAPK and NF-κB inhibitors, whereas the treatment with inhibitors of ERK-MAPK and JNK-MAPK failed to reduce the MMP-2 production. These results were further validated by transfection of SHP-77 cells with pre-miR-125b-5p. The results also showed that the reduced production of MMP-2 by pre-miR-125b-5p-transfected cells was further significantly reduced by the treatment with p38-MAPK and NF-κB inhibitors (*p* < 0.05), whereas the treatment with inhibitors of ERK-MAPK and JNK-MAPK failed to reduce the MMP-2 production (*p* > 0.05) (Figure 6C).

Together, these results consistently demonstrate that p38-MAPK and NF-κB signaling pathways play a key role in regulating miR-125b-5p expression and its downstream target MMP-2 in SHP-77 lung cancer cells, whereas ERK-MAPK and JNK-MAPK pathways appear to be non-contributory.

These findings collectively demonstrate that S100A4-induced RAGE signaling promotes MMP-2 overexpression by directly suppressing hsa-miR-125b-5p through activation of the p38-MAPK and NF-κB (p65/p50) pathways in lung cancer cells.

## 3. Discussion

**Figure 7:**
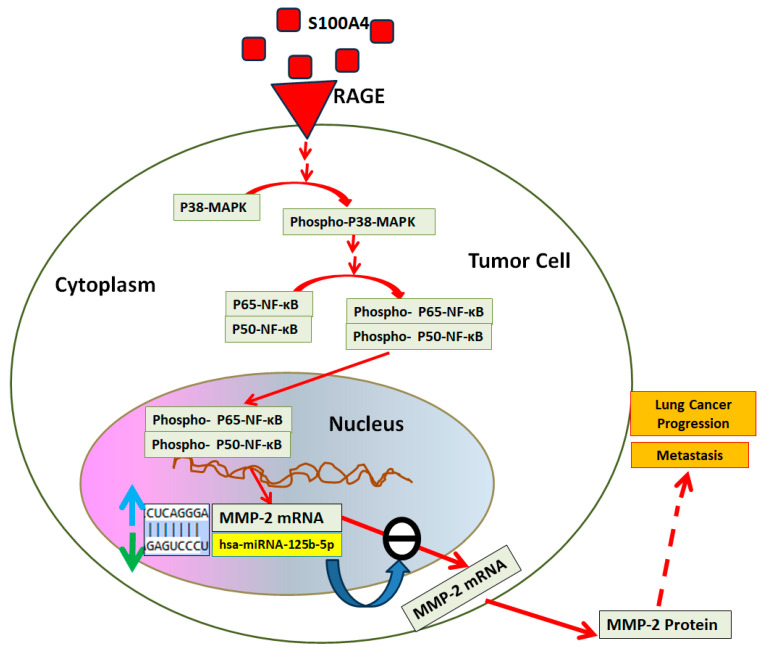
Overview of S100A4-mediated activation of RAGE enhances MMP-2 production by suppressing hsa-miR-125b-5p through p38-MAPK and NF-κB signaling. Schematic representation of S100A4-activated RAGE signaling, p38-MAPK activation, p65/p50-NF-κB activation, hsa-miR-125b-5p down-regulation, and MMP-2 up-regulation in lung cancer cells, highlighting their effects on lung cancer metastasis and progression. Pathways shown in red solid arrow lines were investigated in the study, whereas dashed solid arrow indicated suggestive effects. The blue and green arrows show inverse relation of MMP-2 mRNA and hsa-miR-125b-5p. If MMP-2 mRNA expression is increased than hsa-miR-125b-5p is decreased or vice-versa. The blue arrow represents increased expression of MMP-2 mRNA whereas green arrow represents down-regulation of hsa-miR-125b-5p expression.

Lung cancer remains one of the major causes of cancer-related deaths globally, necessitating an urgent need to explore novel regulatory pathways that facilitate its progression and metastasis. In this study, we provide new insights into the regulatory role of hsa-miR-125b-5p in controlling the expression of MMP-2 through the RAGE-p38MAPK-p65/p50NF-κB axis in human lung cancer cells (Figure 6). These findings underscore the potential of targeting this pathway for therapeutic intervention, as inhibiting hsa-miR-125b-5p may disrupt tumor growth and metastasis. Future research should focus on validating these results in clinical settings and exploring the broader implications of this regulatory mechanism in other cancer types. Our findings reveal that hsa-miR-125b-5p is downregulated upon activation of RAGE by its ligand s100a4, leading to increased MMP-2 expression and enhanced tumor invasion potential (Figure 7).

MMP-2 is an important enzyme involved in extracellular matrix remodeling, which facilitates tumor cell invasion and metastasis. The dysregulation of MMP-2 has been widely associated with the aggressive nature of various malignancies, including lung cancer [4,25]. Our study demonstrates that hsa-miR-125b-5p directly suppresses MMP-2 expression by binding to its 3′UTR, thereby reducing its mRNA stability and translational efficiency. These findings are consistent with earlier reports demonstrating that miRNAs serve as post-transcriptional regulators of MMPs in cancer progression [6,24,26]. The inverse relationship between hsa-miR-125b-5p and MMP-2 expression observed in our study further supports the hypothesis that miRNA-mediated control of MMPs is a key mechanism in tumorigenesis (Figure 7). Several studies demonstrated that MMP-2 overexpression is associated with increased lung cancer invasiveness and poorer patient survival [2,27,28]. This aligns with our findings, indicating that miRNA-targeted regulation of MMP-2 may be an effective strategy for mitigating lung cancer metastasis [29]. In addition, similar regulatory effects of miRNAs on MMP expression have been observed in breast and colorectal cancers [30,31], reinforcing the broader relevance of our findings. RAGE is a multi-ligand receptor known to contribute to inflammation and cancer progression through its interaction with S100A4, a calcium-binding protein associated with metastasis and poor prognosis in lung cancer [32]. Our data indicate that S100A4 stimulation leads to a marked downregulation of hsa-miR-125b-5p, which in turn promotes MMP-2 overexpression. This suggests that RAGE activation negatively regulates hsa-miR-125b-5p expression, thereby enhancing the invasive capabilities of lung cancer cells. Previous research has demonstrated that the RAGE-S100A4 axis is involved in multiple cancers, including pancreatic and colorectal cancers, by modulating miRNA expression and enhancing metastatic properties [33,34,35]. Our findings add to this expanding body of information, further elucidating the interplay between inflammatory signaling and miRNA-mediated tumor regulation.

MAPKs and NF-κB are major signaling pathways implicated in inflammatory and oncogenic processes [10,23,36]. Our results indicate that inhibition of p38MAPK using SB202190 restores hsa-miR-125b-5p levels, leading to decreased MMP-2 expression. This suggests that p38MAPK acts as a negative regulator of hsa-miR-125b-5p in lung cancer cells, consistent with studies indicating that MAPK signaling contributes to the dysregulation of tumor-suppressive miRNAs [22]. NF-κB, a transcription factor known for its role in promoting cancer cell survival and invasion, was found to be significantly activated upon S100A4 stimulation in this study, which demonstrated that NF-κB inhibition via Bay-11-7082 rescues hsa-miR-125b-5p expression, thereby reducing MMP-2 levels. This aligns with prior research highlighting the crosstalk between NF-κB and miRNA networks in controlling MMP expression and tumor progression [37]. Importantly, it is also reported that NF-κB activation leads to the suppression of tumor-suppressive miRNAs in lung cancer and various other cancer [27,38,39], further reinforcing our findings. The observations made in A549 lung cancer cells were substantiated by experiments in SHP-77 cells, thereby strengthening the reliability of the findings. Using pharmacological inhibition, the contribution of MAPK and NF-κB pathways was systematically assessed. Inhibition of p38-MAPK and NF-κB consistently restored miR-125b-5p expression in S100A4-stimulated SHP-77 cells, whereas blockade of ERK-MAPK or JNK-MAPK failed to induce similar effects. These results were corroborated through both loss-of-function (anti-miR-125b-5p) and gain-of-function (pre-miR-125b-5p mimic) approaches, further confirming the specificity of the p38-MAPK and NF-κB pathways in regulating miR-125b-5p. The downstream consequences of this regulation were evident at the level of MMP-2 expression. Pretreatment with p38-MAPK or NF-κB inhibitors led to significant suppression of MMP-2 mRNA and protein secretion, while inhibition of ERK-MAPK or JNK-MAPK remained ineffective. Importantly, in anti-miR-125b-5p-transfected cells, excessive MMP-2 production was markedly reduced following inhibition of p38-MAPK or NF-κB, but not by ERK-MAPK or JNK-MAPK inhibitors. Conversely, in pre-miR-125b-5p-transfected cells, already reduced MMP-2 levels were further diminished by p38-MAPK or NF-κB inhibition, again underscoring their critical role. Taken together, these results provide consistent evidence across different experimental conditions that p38-MAPK and NF-κB signaling are the principal regulators of the miR-125b-5p/MMP-2 axis in SHP-77 lung cancer cells. By contrast, ERK-MAPK and JNK-MAPK pathways appear to be non-essential in this regulatory mechanism. These findings highlight the potential therapeutic significance of selectively targeting the p38-MAPK and NF-κB pathways in modulating miR-125b-5p function and its downstream effector MMP-2 in lung cancer progression.

Our study underscores the potential of hsa-miR-125b-5p as a treatment target for lung cancer. Restoring hsa-miR-125b-5p levels through miRNA mimics or inhibitors of p38MAPK and NF-κB pathways could offer a novel strategy to suppress MMP-2-driven metastasis. Given that miRNA-based therapies are currently under investigation in clinical settings, our findings contribute significantly to the current knowledge supporting the development of miRNA-targeting interventions in cancer treatment [40,41]. Further research is warranted to explore the in vivo relevance of our findings using lung cancer xenograft models. Moreover, investigating the interplay between hsa-miR-125b-5p and other oncogenic pathways may provide deeper insights into its regulatory mechanisms. Finally, assessing the clinical significance of hsa-miR-125b-5p and MMP-2 expression in lung cancer patients could help validate our results and pave the way for biomarker-based diagnostic and prognostic applications.

## 4. Methods

### 4.1. Bioinformatics Analysis

The TargetScan online tool (http://www.targetscan.org/, accessed on 12 November 2024) was used to identify putative microRNA binding sites within the 3′ untranslated region (3′UTR) of MMP-2. The computational analysis for predicting miRNA target sequences followed established protocols from previous studies [42].

### 4.2. Lung Cancer Cell Culture and S100A4 Treatment

Human lung cancer (LC) A549 and SHP-77 cell lines from the American Type Culture Collection (Rockville, MD, USA) were grown in RPMI 1640 media with 10% fetal bovine serum (FBS) and 1% penicillin-streptomycin as described previously [43]. A549 cells were kept at 37 °C in a humid environment with 5% CO_2_. After reaching 70–80% confluence, cells were serum-starved overnight and treated with S100A4 (1 µg/mL) for varying durations. Untreated cells served as negative controls.

### 4.3. RNA Extraction, cDNA Synthesis, and qPCR

MirVana miRNA Isolation Kit (Ambion, Austin, TX, USA) was used to isolate total RNA, including microRNA. The Superscript First-Strand cDNA Synthesis Kit (Applied Biosystems, Waltham, MA, USA) was used to generate complementary DNA (cDNA). TaqMan assays were used to perform quantitative PCR (qPCR) using StepOne Real-Time PCR System (Life Technologies, Thermo Fisher Scientific, Carlsbad, CA, USA). To normalize mRNA and miRNA expression, GAPDH and RNU6B were utilized as reference genes. The ΔΔCT method was applied for relative quantification as previously described [44,45].

### 4.4. Western Blot Analysis and Densitometry

Western blotting was used to measure MMP-2 and β-actin levels in cell culture supernatants as previously described [46]. Proteins were separated using SDS-PAGE, transferred to PVDF membranes, and then probed with MMP-2-specific antibodies. Protein was visualized, and densitometry analysis was carried out with UNSCAN-IT software. Version 7.0 (Silk Scientific, Provo, UT, USA).

### 4.5. Luciferase Reporter Assay

Lung cancer cells were co-transfected with luciferase reporter plasmids and either pre-miR-125b-5p or anti-miR-125b-5p (Qiagen, Valencia, CA, USA; Ambion, Austin, TX, USA) using Qiagen’s HiPerfect Transfection Reagent as previously described [7]. Cells were transfected with reporter plasmids alone or in combination with a negative control microRNA (miR-NC). The Dual-Luciferase Reporter Assay System (Promega, Madison, WI, USA) was utilized for luciferase activity measurement 72 h after transfection. Firefly luciferase activity was normalized to Renilla luciferase activity according to the manufacturer’s procedure (Promega).

### 4.6. Cell Transfection and Inhibitor Treatments

A549 and SHP-77 cells were transfected with either pre-miRNAs or anti-miRNAs (100 nM; Ambion/Qiagen) using Qiagen HiPerfect Transfection Reagent. After 72 h, transfected cells were pretreated with inhibitors targeting MAPKs or NF-κB for 2 h before stimulation with S100A4 for 30 min to 24 h. Following treatment, RNA and protein were extracted for downstream analysis.

### 4.7. Nuclear Extract Preparation and NF-κB Activity Assay

To examine the impact of hsa-miR-125b-5p on S100A4-mediated activation of p65-NF-κB and p50-NF-κB, A549 cells were transfected with either anti-miR-125b-5p or miR-NC (100 nM) and then treated or left untreated with S100A4 (1 µg/mL) for 30 min. LC cells were rinsed with ice-cold PBS, collected by centrifugation at 1500× *g* for 5 min at 4 °C, and processed for nuclear protein extraction following standard protocols [27]. Equal amounts of nuclear protein were used to assess p65-NF-κB and p50-NF-κB activation or destabilization using a Transcription Factor ELISA Kit (catalog # ab133128, Abcam, Waltham, MA, USA) according to the manufacturer’s instructions.

### 4.8. MMP-2 Quantification

MMP-2 levels in culture medium of lung cancer cells were quantified by human MMP-2 highly specific Sandwich ELISA (catalog# ab100606, Abcam, Cambridge, UK), according to the manufacturer’s protocols.

### 4.9. Statistical Analysis

Statistical analyses were performed using GraphPad Prism 5 (San Diego, CA, USA). Data were analyzed using either one-way ANOVA followed by Tukey’s post hoc test or two-way ANOVA with Bonferroni corrections. A *p*-value of <0.05 indicated statistical significance.

## 5. Conclusions

This study determined a novel regulatory mechanism by which hsa-miR-125b-5p modulates MMP-2 expression via RAGE-p38MAPK-NF-κB signaling in lung cancer cells. RAGE activation inhibits hsa-miR-125b-5p, which increases MMP-2 expression and promotes tumor invasion. Our findings are supported by multiple studies across different cancer types, underscoring the significance of miRNA-mediated regulation in cancer metastasis. Furthermore, these insights may lead to the development of targeted therapies aimed at disrupting the RAGE-p38MAPK-NF-κB pathway, potentially mitigating the invasive properties of lung cancer. Continued research in this area could unveil additional miRNAs that play critical roles in tumor progression, offering new avenues for intervention. Targeting this pathway represents a promising therapeutic approach to mitigate lung cancer progression. Future studies focusing on in vivo validation and clinical translation will be essential to harness the full potential of miRNA-based interventions in lung cancer therapy.

## Data Availability

All data and materials used in this study are available from the corresponding author and will be provided upon reasonable request.
